# Absence of Tau triggers age‐dependent sciatic nerve morphofunctional deficits and motor impairment

**DOI:** 10.1111/acel.12391

**Published:** 2016-01-08

**Authors:** Sofia Lopes, André Lopes, Vítor Pinto, Marco R. Guimarães, Vanessa Morais Sardinha, Sara Duarte‐Silva, Sara Pinheiro, João Pizarro, João Filipe Oliveira, Nuno Sousa, Hugo Leite‐Almeida, Ioannis Sotiropoulos

**Affiliations:** ^1^ Life and Health Sciences Research Institute (ICVS)School of Health SciencesUniversity of MinhoCampus Gualtar4710‐057BragaPortugal; ^2^ ICVS/3B's – PT Government Associate LaboratoryBraga/GuimarãesPortugal

**Keywords:** Tau, motor deficits, peripheral nerve, myelination, nerve conduction, knockout

## Abstract

Dementia is the cardinal feature of Alzheimer's disease (AD), yet the clinical symptoms of this disorder also include a marked loss of motor function. Tau abnormal hyperphosphorylation and malfunction are well‐established key events in AD neuropathology but the impact of the loss of normal Tau function in neuronal degeneration and subsequent behavioral deficits is still debated. While Tau reduction has been increasingly suggested as therapeutic strategy against neurodegeneration, particularly in AD, there is controversial evidence about whether loss of Tau progressively impacts on motor function arguing about damage of CNS motor components. Using a variety of motor‐related tests, we herein provide evidence of an age‐dependent motor impairment in *Tau−/−* animals that is accompanied by ultrastructural and functional impairments of the efferent fibers that convey motor‐related information. Specifically, we show that the sciatic nerve of old (17–22‐months) *Tau−/−* mice displays increased degenerating myelinated fibers and diminished conduction properties, as compared to age‐matched wild‐type (*Tau+/+*) littermates and younger (4–6 months) *Tau−/−* and *Tau+/+* mice. In addition, the sciatic nerves of *Tau−/−* mice exhibit a progressive hypomyelination (assessed by g‐ratio) specifically affecting large‐diameter, motor‐related axons in old animals. These findings suggest that loss of Tau protein may progressively impact on peripheral motor system.

## Introduction

Clinical presentation of Alzheimer's disease (AD) is complex and extends well beyond the cognitive impairments that characterize this disorder (Duker *et al*., 2012). Alterations in facial expression, gait and posture, and manifestations of rigidity, bradykinesia, and tremor are found in late AD stages although mounting evidence suggests that motor problems emerge long before any recognizable sign of AD (Wilson *et al*., 2000; Scarmeas *et al*., 2004; Buchman & Bennett, 2011). Tau abnormal hyperphosphorylation and subsequent malfunction are postulated as crucial mechanisms in AD neuronal dysfunction where hyperphosphorylated and/or aggregated (insoluble) forms of Tau exhibit neurodegenerative action(s) that also interfere with normal Tau, sequestering and reducing soluble Tau forms (Ksiezak‐Reding *et al*., 1988; Zhukareva *et al*., 2003). These lesions are mainly found at different areas of CNS such as hippocampus and cortex although some studies also demonstrate the presence of Tau deficits in peripheral nervous system (PNS; e.g., autonomic ganglia and sciatic nerves Bohl *et al*., 1997; Holzer *et al*., 1999). While evidence suggests that Tau reduction can block AD pathology progression (Roberson *et al*., 2007), indicating that Tau‐targeted strategies might be of interest for AD therapy (Gotz *et al*., 2012), the safety and/or potential side effect(s) of these approaches is not well studied.

Tau genetic deletion seems to be well tolerated by young animals as the majority of *Tau−/−* models do not exhibit behavioral or microtubule alterations (Dawson *et al*., 2001; reviewed by Ke *et al*., 2012). However, chronic loss of Tau has been described to result in subtle or mild motor deficits in increasingly aged animals (reviewed by Gotz *et al.,* 2013). Indeed, one study revealed loss of substantia nigra (SN) dopaminergic neurons in middle‐aged *Tau−/−* animals (Lei *et al*., 2012), while in another study, using aged *Tau−/−* of the same strain, similar motor deficits were shown but in a SN‐/dopamine‐independent manner (Morris *et al*., 2013), raising uncertainty on the underlying mechanisms of the motor deficits in *Tau−/−* animals. Surprisingly, the involvement of the PNS has not been assessed in any of the previous studies given the fact that Tau reduction is found in peripheral nerves (e.g., sciatic nerve) of patients with AD (Holzer *et al*., 1999). This study aimed therefore to monitor the impact of chronic loss of Tau protein in PNS efferents, primary compartment of motor circuitry, and motor performance using a battery of behavioral tests analyzing motor function in both young (4–6 months) and old (17–22 months) *Tau−/−* mice combined with a systematic morphofunctional analysis of their sciatic nerve.

## Results

### Chronic lack of Tau results in motor deficits in old animals

**Figure 1:**
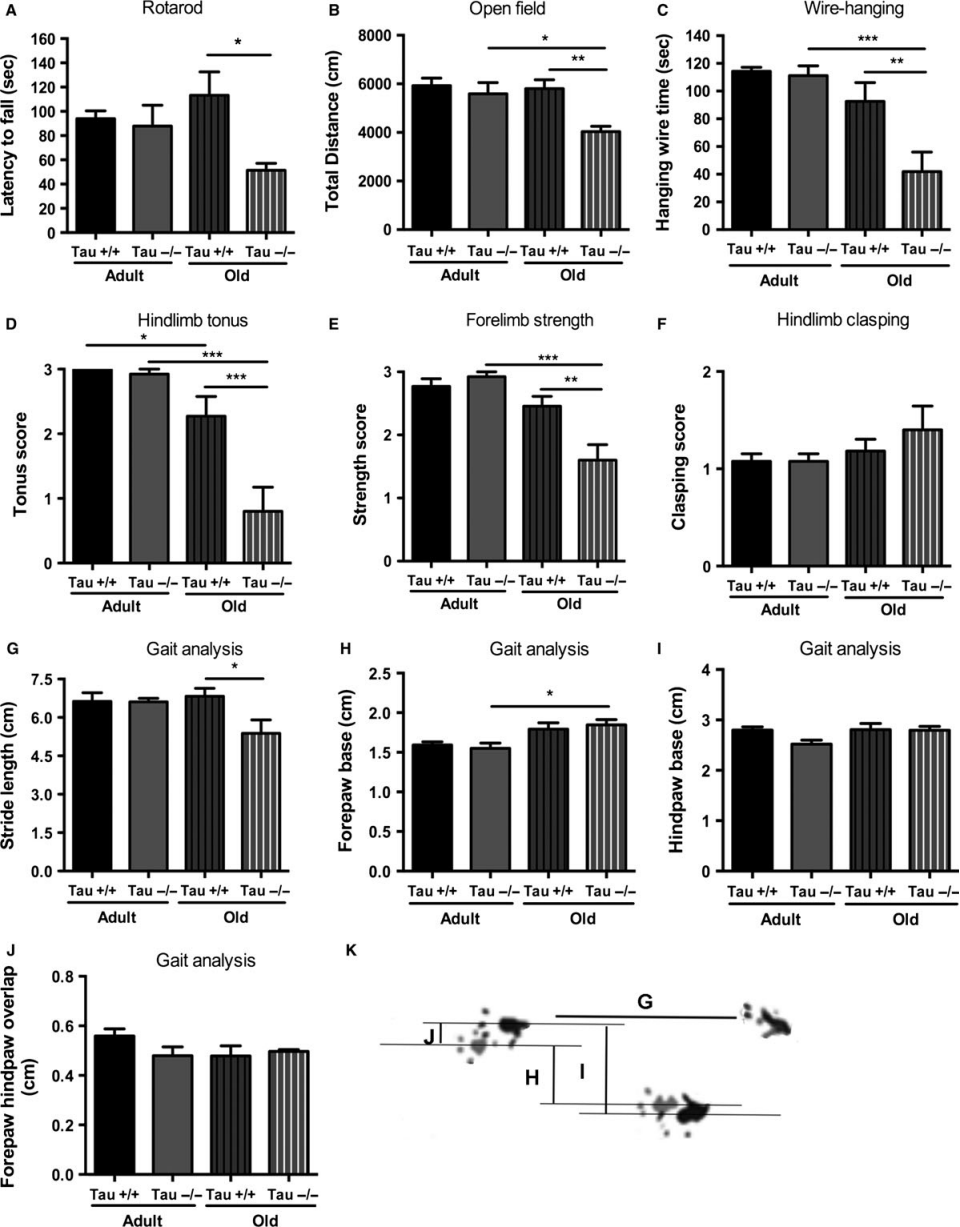
Impaired motor behavior of old *Tau−/−* animals. Behavioral screening of motor functions revealed that lack of Tau protein led to an age‐dependent motor dysfunction. (A) Rotarod test showed that old, but not adult, *Tau−/−* animals exhibited reduced latency to fall when compared with *Tau+/+* reflecting motor impairment. (B) Total distance travelled in open field was reduced in old *Tau−/−* when compared with old *Tau+/+* and adult *Tau−/−* animals indicating reduced locomotor activity. (C) Wire‐hanging test assessments showed that old *Tau−/−* animals differed from old *Tau+/+* and adult *Tau−/−* presenting significantly less hanging time. D‐E) Hindlimb tonus resistance (D) and forelimb strength (E) were severely reduced in old *Tau−/−* animals compared with old and adult *Tau+/+*. (F) No changes in hindlimb clasping score between animals of both ages and genotypes. (G–J) Footprinting‐based gait analysis showed that, in contrast to adult animals, old *Tau−/−* present reduced stride length when compared to old *Tau+/+* (G), while no other changes were found between animals of two genotypes. (K) Representative image of animal footprints which were used to calculate the different gait parameters, for example, stride length *(G), forepaw base (H), hindpaw base (I), and forepaw/hindpaw overlap (J). All numeric data are presented as mean ± SEM; *p < 0.05; **p < 0.01; ***p < 0.001.

To clarify the discrepancies of previous studies regarding the age‐dependent motor deficits in *Tau−/−* animals (cf. Lei *et al*., 2012 and Morris *et al*., 2013), we performed a battery of motor‐related behavioral tests in adult (4–6 months old) and old (17–22 months old) male *Tau−/−* and *Tau+/+* animals. Motor function/coordination and locomotor activity were assessed in the rotarod and open‐field tests, respectively. As shown in Fig. 1A, the accelerating protocol of rotarod test showed that old *Tau−/−* animals presented reduced latency to fall, indicating an impairment in motor coordination. Two‐way ANOVA rendered an overall genotype effect [*F*_1,37_ = 4.050, p = 0.021], where old *Tau−/−* exhibit a motor impairment when compared with old *Tau+/+* (p = 0.019). In the open‐field arena, we found a *Genotype x Aging* interaction of total area travelled by the animals [*F*_1,31_ = 4.339, p = 0.045] (Fig. 1B). Further analysis revealed that old *Tau−/−* travelled significantly less than their age‐matched *Tau+/+* (p = 0.005) as well as than adult *Tau−/−* animals (p = 0.019). As potential body weight differences among groups could affect the assessment of the above motor‐related parameters, we monitor body weight of these animals (adult *Tau+/+*: 27.876 ± 0.95; adult *Tau−/−*: 27.879 ± 0.53; old *Tau+/+*: 34.943 ± 0.60; old *Tau−/−*: 36.215 ± 1.67) finding no overall *Genotype* ([*F*_1,37_ = 0.4187, p = 0.52]), but a significant *Aging* overall effect ([*F*_1,37_ = 61.15, p < 0.0001]). Further analysis showed that both old *Tau+/+* and *Tau−/−* animals exhibit higher body weight than their younger counterparts (p < 0.0001 for both genotypes). For monitoring muscle strength, we use wire‐hanging test where a *Genotype x Aging* interaction [*F*_1,31_ = 5.768, p = 0.022] was found; old *Tau−/−* present significantly less hanging time than old *Tau+/+* (*P* = 0.008) as well as when compared with adult *Tau−/−* (p = 0.0001) suggesting compromised muscle strength (Fig. 1C). Furthermore, monitoring separately hindlimb and forelimb performance by hindlimb tonus and forelimb grid strength tests, we found a *Genotype x Aging* interaction (for tonus *F*_1,38_ = 11.97, p = 0.001; for strength *F*_1,38_ = 12.14, p = 0.001) represented by diminished hindlimb tonus resistance and reduced forelimb strength in old *Tau−/−* animals when compared to old *Tau+/+* (p_tonus_ = 0.0003 and p_strength_ = 0.004, respectively; Fig. 1D,E); note that this difference was not found in adult animals (p_tonus_ = 0.98 and p_strength_ = 0.80, respectively) in line with the findings of the above tests used in this study. In addition, no significant changes among groups were found in hindlimb clasping score (Fig. 1F). Moreover, for extra monitoring of motor coordination and gait, we performed footprinting‐based analysis showing a *Genotype x Aging* interaction in the stride length of animals (*F*_1,23_ = 4.69, p = 0.04) where, in contrast to adult animals, old *Tau−/−* presented a decreased stride length when compared to old *Tau+/+* (p = 0.03) (Fig. 1G). In addition, we found an *Aging* main effect on forepaw base (*F*_1,23_ = 13.01, p = 0.001) where old *Tau−/−* animals presented an increase in this parameter compared to adult *Tau−/−* animals (p = 0.04) (Fig. 1H). No significant differences were found in other parameters of gait analysis such as hindpaw base and forepaw–hindpaw overlap.

### Tau ablation alters sciatic nerve structure and function by aging

**Figure 2:**
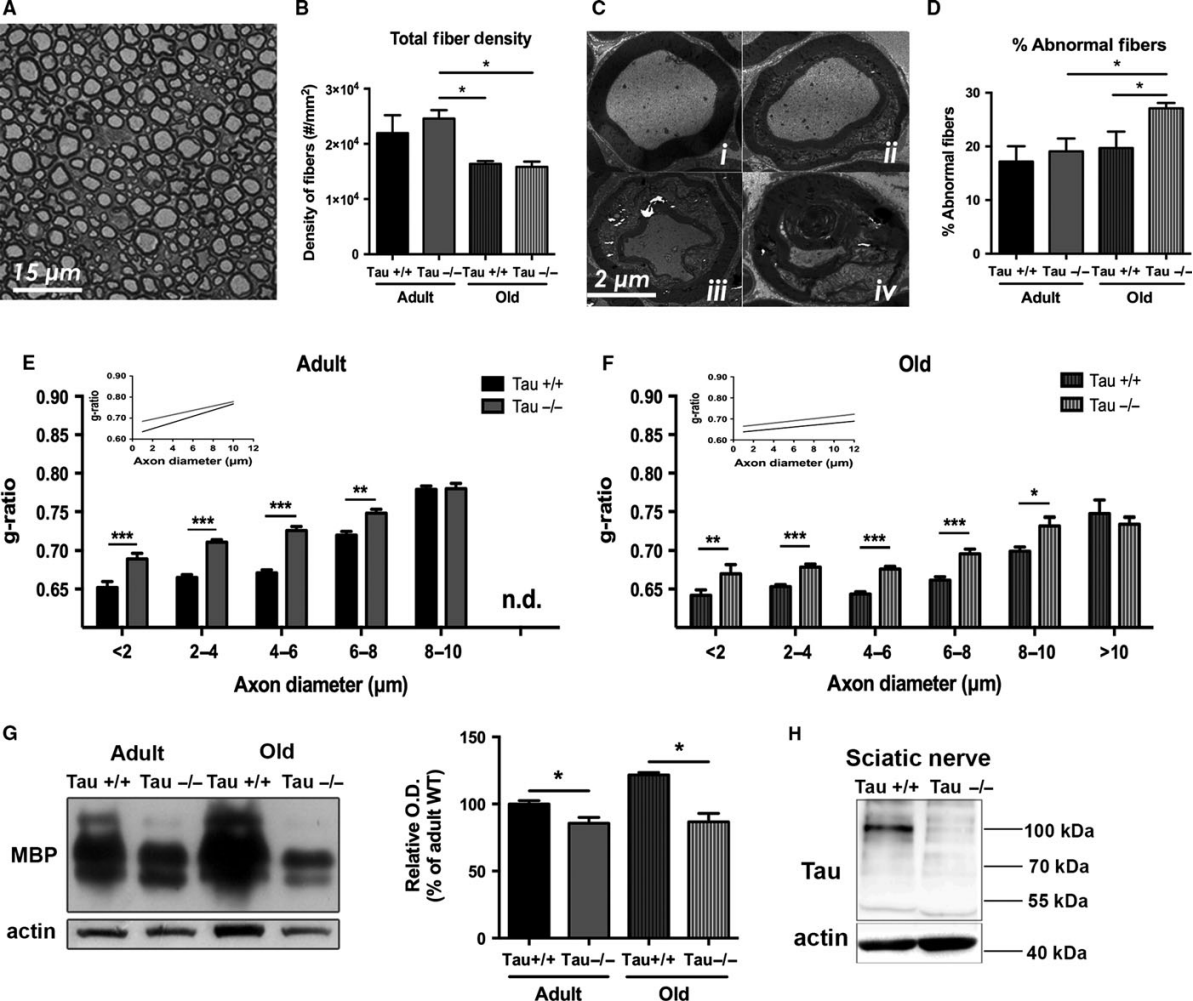
Ultrastructural analysis of *Tau−/−* and *Tau+/+* sciatic nerves. (A, B) Representative light microscopy photograph of ppd staining of sciatic nerve (A) and quantification of fibers density (B) showing an age‐dependent reduction in both *Tau−/−* and *Tau+/+*. (C) TEM analysis images of sciatic nerve showing normal (i) and degenerating (ii, iii) myelinated fibers with obvious myelin anomalies while severely damaged axons (iv) were very rarely found in all groups. (D) Old *Tau−/−* animals present a significant increase in percentage of degenerating fibers when compared with old *Tau+/+* animals. (E, F) Detailed evaluation of g‐ratio [axon diameter/(axon diameter + myelin thickness)] per axon diameter category in adult (E) and old (F) animals. There is an age‐dependent g‐ratio reduction (hypomyelination) in Tau*−/−* vs. *Tau+/+* starting from axons of small and middle diameter (nonmotor related) in adult animals (E) and extending to large‐diameter (8–10 μm), motor‐related axons in old Tau*−/−* (F). Specifically, compared to adult *Tau+/+* ones, adult *Tau−/−* animals exhibited increased g‐ratio in all fiber categories except that of 8–10 μm, while in old *Tau−/−*, this category g‐ratio was also affected (E). Insets represent regression lines of g‐ratio growth in *Tau−/−* (red) and *Tau+/+* (black) of adult (D) and old (E) animals where the regression lines for adult, but not old, animals are converging at the large‐diameter axons, indicating no difference between g‐ratio of *Tau−/−* and *Tau+/+* of this fiber category. G‐H) Western blot analysis showed reduced myelin basic protein (MBP) levels in Tau−/− sciatic nerves (G) in line with the g‐ratio analysis as well as absence of the characteristic high molecular weight Tau (HMW‐Tau; 110 kDa) (H) in sciatic nerves of Tau−/− animals. Data are presented as mean ± SEM; *p < 0.05; **p < 0.01; ***p < 0.001.

As *Tau−/−* mice are shown to exhibit motor deficits, it was of interest to monitor the impact of Tau ablation on the peripheral component of motor circuit; for that purpose, we studied the sciatic nerve, in the same mouse line and background (C57BL/6; Dawson *et al.,* 2001) used in previous studies describing motor deficits after Tau ablation (Morris *et al*., 2013; Lei *et al*., 2014). It is important to note that the sciatic nerve is known to exhibit important morphological and functional changes with aging (Melcangi *et al*., 2000). Thus, we monitored myelinated (A‐fibers) density in p‐phenylene diamine (PPD)‐stained sections. In agreement with previous studies (Ceballos *et al*., 1999), two‐way ANOVA showed an aging effect [*F*_1,16_ = 11.71, p = 0.0038] with aged animals exhibiting reduced density of myelinated fibers independently of genotype (p < 0.05 for both genotypes) (Fig. 2A,B). Furthermore, electron micrographs of the sciatic nerves revealed, in addition to normal myelinated axons (Fig. 2C*i*), the presence of dysmorphic myelin sheaths as shown in Fig. 2C*ii–*i*v*. Thus, we also evaluated the percentage of myelinated fibers that exhibit the above abnormalities in their myelin sheath finding an overall aging effect [*F*_1,16_ = 7.41, p = 0.015] and an overall genotype effect [*F*_1,16_ = 5.58, p = 0.031]. Additional analysis showed a significant increase in the percentage of degenerating myelinated fibers in old *Tau−/−* animals when compared with old Tau*+/+* animals (p = 0.049) and adult *Tau−/−* (p = 0.0289) (Fig. 2D). In the next step of our analysis, we compared myelin sheath thickness of fibers of sciatic nerves by electron microscopy determining the g‐ratio, an index of myelination independent of axonal diameter (Michailov *et al*., 2004). A total of 2885 axons were sampled and classified according to axon diameter. *Tau−/−* axons exhibited increased g‐ratio indicating reduced myelination while two‐way ANOVA per category exhibited an overall *Genotype* effect in all categories [*F*_1,270_ = 23.891, p < 0.001 (0–2 μm); *F*_1,1390_ = 99.885, p < 0.001 (2–4 μm); *F*_1,705_ = 132.891, p < 0.001 (4–6 μm); *F*_1,372_ = 39.379, p < 0.001 (6–8 μm); *F*_1,143_ = 9.060, p = 0.02 (8–10 μm)] and an age effect [*F*_1,1390_ = 31.672 (2–4 μm); *F*_1,705_ = 74.302 (4–6 μm); *F*_1,372_ = 100.001 (6–8 μm); *F*_1,143_ = 30.694 (8–10 μm); p < 0.001] in all categories except 0–2 μm class of axons (*F*_1,270_ = 3.256; p = 0.072) (Fig. 2E,F). Large‐diameter (8–10 μm) axons g‐ratio (motor‐related) was different between old (p < 0.001), but not adult, *Tau+/+* and *Tau−/−*. This g‐ratio variation in large‐diameter axons between *Tau−/−* and *Tau+/+* of the two ages is also depicted in the regression lines of g‐ratio growth in *Tau−/−* (grey) and *Tau+/+* (black) of adult and old animals (inserted micrographs in Fig. 2E and 2F, respectively), where the regression lines for adult, but not old, animals are converging at the large‐diameter axons indicating no difference between g‐ratios of *Tau−/−* and *Tau+/+* of this fiber category. The above ultrastructural hypomyelination findings in sciatic nerves of *Tau−/−* animals were further confirmed by reduced protein levels of myelin basic protein (MBP) (Fig. 2G).

**Figure 3:**
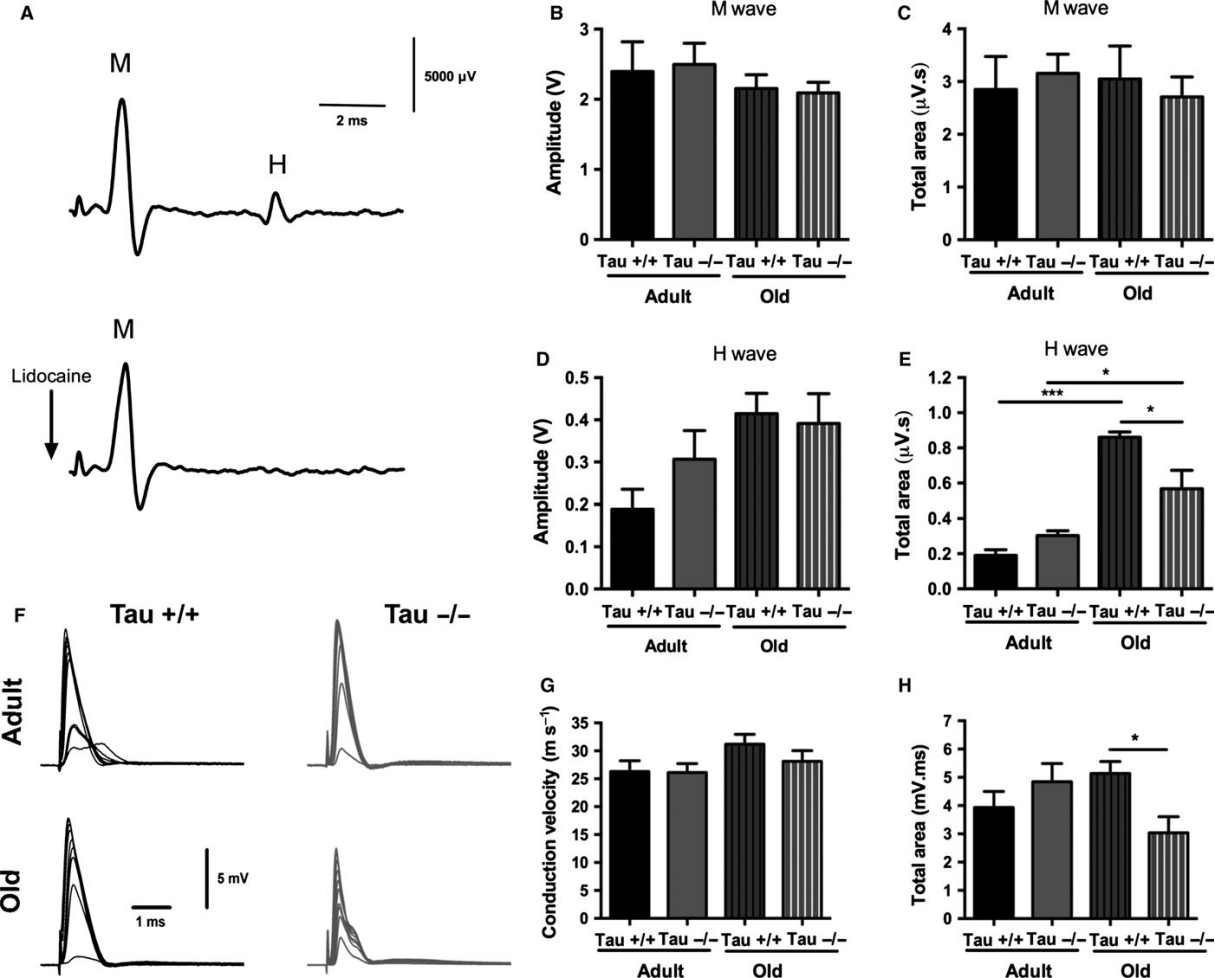
Tau ablation results in age‐dependent sciatic nerve malfunction. (A) Representative traces of *in vivo* electrophysiological recordings of compound muscle (M) and reflex (H) nerve action potentials (upper panel); lidocaine injection locally at the sciatic resulted in loss of H‐wave without affecting the muscle M‐wave (lower panel). (B, C) No differences in both amplitude (B) and total response area (C) of M‐waves between animals of both genotypes and ages. (D) Age‐dependent increase in amplitude of H‐waves (*Aging* main effect, see also results), but no difference between *Tau−/−* and *Tau+/+* animals. (E) Old *Tau−/−* exhibited significantly reduced total response area compared to old *Tau+/+* animals, while this difference was not found in adult animals. In addition, there was an age‐dependent difference in both *Tau+/+* and *Tau−/−* animals. (F) Representative traces of current‐clamp recordings of sciatic nerve compound action potentials in adult and old *Tau+/+* and *Tau−/−* mice. Sciatic nerves received repeated stimuli with increasing amplitudes pulses until saturation. Stimulation artifacts were truncated (conduction distance: adult *Tau+/+*: 5.5 mm; adult *Tau−/−*: 9.6 mm; old *Tau+/+*: 8.0 mm; old *Tau−/−*: 9.8 mm); traces are averages of five consecutive recordings. (G, H) While no differences in conduction velocity (m s^−1^) between *Tau+/+* and *Tau−/−* were found (G), total area under the curve (mV.ms) of old *Tau−/−* was significantly reduced compared to old *Tau+/+* animals indicating sciatic nerve malfunction (H). Data presented as mean ± SEM; p < 0.05.

Next, we monitored *in vivo* compound muscle (M) and reflex (H) nerve action potentials in hindpaw of anesthetized animals (Fig. 3A–E). As shown in Fig. 3B,C, there were no differences between amplitudes or total response area of direct muscle action potentials—M‐wave—between *Tau+/+* and *Tau−/−* on both ages. Regarding the H‐waves which represent the reflex response that travels *via* the sciatic nerve (sensitive to lidocaine local sciatic application; Fig. 3A), two‐way ANOVA showed an *Aging* main effect [*F*_1,17_ = 6.701, p = 0.018] on the amplitude but no changes between *Tau−/−* and *Tau+/+* animals of both ages (Fig. 3D). Moreover, assessment of the total response area exhibited a significant reduction in old *Tau−/−* animals when compared to old *Tau+/+* (p = 0.02; Fig. 3E); note that this difference was not found in adult animals (p = 0.64). We also found a *Genotype x Aging* interaction effect [*F*_1,17_ = 9.40, p = 0.007] as well as an aging main effect [*F*_1,17_ = 49.78, p < 0.0001]. Based on the morphological alterations of sciatic nerve described above, we next monitored nerve function by *ex vivo* recordings in isolated sciatic nerves, as previously described (Pinto *et al*., 2008) (Fig. 3F). Two‐way ANOVA revealed no differences in conduction velocity of A‐fibers among both ages and genotypes (Fig. 3G). However, assessment of the total response area revealed a significant *Genotype x Aging* interaction [*F*_1,31_ = 6.715, *P* = 0.0144]. Particularly, we observed that sciatic nerves from old *Tau−/−* animals presented a decreased conduction capacity when compared with old *Tau+/+* animals (*P* = 0.03) (Fig. 3H), indicating diminished overall response ability for old *Tau−/−* sciatic nerves; the same was not valid for adult animals again providing further evidence about the age‐dependent impact of Tau loss on sciatic nerve.

## Discussion

Tau is a soluble cytoskeletal protein that is expressed mainly in neurons and in glial cells, of both CNS and PNS; it is implicated in various cellular processes such as microtubule stabilization, axonal maintenance, and transport. Tau malfunction is also causally related to AD pathology in where aberrant, hyperphosphorylated and/or aggregated, forms of Tau are found in the CNS and in the PNS (Holzer *et al*., 1999). Accordingly, recent evidence suggests Tau protein as the final executor of AD neuronal dysfunction (Roberson *et al*., 2007; Ittner & Gotz, 2011) and, therefore, anti‐Tau therapies emerged as potential strategies against AD (Frost *et al*., 2015); however, their safety or potential risks have not been defined yet.

Evidence from different *Tau−/−* models suggests that absence of Tau is well tolerated by young/adult animals probably due to the occurrence of compensatory mechanisms (e.g., increase in MAP1A, another microtubule‐associated protein) (Dawson *et al*., 2001; Ma *et al*., 2014). In contrast, aged *Tau−/−* seem to exhibit mild motor deficits albeit the effect of Tau depletion on motor performance by aging remains controversial and unclear. A study from Ikegami *et al*. (2000) reports signs of muscle weakness (tested by wire‐hanging) and reduced balance coordination in 2‐month‐old *Tau−/−*, but Morris *et al*. (2011) showed normal motor function of young (3–5 months old) and adult animals of another Tau*−/−* mouse line in a variety of motor tests (B6 background; Dawson *et al*. (2001)) in agreement with this study findings. Moreover, the same team found mild motor deficits in middle‐aged (12–15 months old) *Tau−/−* animals using rotarod and pole tests but, unexpectedly, these motor deficits were not clearly evident in old (21–22 months old) *Tau−/−* (Morris *et al*., 2013). In contrast, we observed clear motor deficits in old (17–22 months old) *Tau−/−* using the rotarod, wire‐hanging, and open‐field tests and these deficits were not correlated with body weight. In addition, our study offers additional evidence of motor dysfunction showing that, in line with our wire‐hanging data, old *Tau−/−* animals also exhibit reduced forelimb (grip) strength and diminished hindlimb tonus resistance compared to old wild‐types but they exhibit no deficits in gross aspects of motor function/balance as assessed by hindlimb clasping tests (see Fig. 1) and negative geotaxis (data not shown). Interestingly, reduced motor strength and coordination was also shown in 13‐ to 17‐month‐old animals lacking 4R‐Tau, suggesting the potential role of 4RTau in the age‐dependent development of motor deficits (Gumucio *et al*., 2013).

The mechanisms underlying the impaired motor behavior in the absence of Tau in aged individuals are not entirely understood with contradictory findings being reported. Lei *et al*. (2012), for instance, reported an age‐related loss of SN dopaminergic neurons in middle‐aged *Tau−/−* animals. In contrast, a recent study on the same *Tau−/−* line failed to observe any major dopaminergic loss in different CNS motor components of old *Tau−/−* mice (Morris *et al*., 2013). While both studies showed motor deficits, Lei *et al*. (2012) showed nigrostriatal loss in middle‐aged Tau−/− mice of C57BL/6/SV129 background, while our study and the study of Morris *et al*. (2013) found no nigrostriatal neuronal loss in old mice with C57BL/6 background (data not shown). Furthermore, in a later study, Lei *et al*. (2014) showed that motor deficits of old *Tau−/−* animals do not depend on background or have a sex‐dependent profile. In line with it, despite the fact that our study has used male animals and Morris study (2013) used a mixed (male and female) cohort, both studies exhibit motor deficits providing further support of no gender influence in the old *Tau−/−* motor deficits.

While a number of CNS areas have been implicated in the emergence of AD‐associated motor deficits, little attention has been devoted to PNS primary efferents. Previous evidence suggests that the sciatic nerves of patients with AD, but not of age‐matched healthy individuals, exhibit reduced Tau levels (Holzer *et al*., 1999). In our study, we demonstrate that chronic loss of Tau protein results in sciatic nerve morphofunctional deficits which includes increased percentage of degenerating fibers, hypomyelination of large‐diameter, motor‐related fibers, and diminished conduction properties in old, but not young, *Tau−/−* sciatic nerve. While other mechanisms cannot be excluded, the aforementioned sciatic nerve deficits may critically contribute to motor deficits found in old *Tau−/−* as fine‐tuning of myelin sheath thickness and formation is important for maintenance and proper function of motor fibers. In addition, while no amplitude differences in muscle action potentials of *Tau−/−* were found, further studies are needed to clarify the impact of loss of Tau on neuromuscular junction by aging as Tau‐related pathology in motor neurons has been shown to have neuromuscular junction malfunction and motor deficits in AD Tg models (Zhang *et al*., 2005; Ubhi *et al*., 2007).

Despite the fact that axon–Schwann cell interactions are critical for nerve function and maintenance, the underlying mechanisms of maintaining normal nerve and Schwann cell structure and function remain poorly understood. Tau is expressed in both CNS and PNS, but the low molecular weight isoforms of Tau protein that are expressed in adult CNS differ from high molecular weight (HMW) Tau isoforms mainly found in PNS (e.g., sciatic nerves) but also in optical nerves (Sato‐Yoshitake *et al*., 1989; Georgieff *et al*., 1991; Nothias *et al*., 1995). The expression of HMW tau isoforms may confer increased stabilization and spacing of microtubules (Frappier *et al*., 1994; Boyne *et al*., 1995) but yet, our knowledge about Tau function in PNS is very limited. While previous cell‐based evidence suggests the involvement of Tau in myelination of oligodendrocytes, the current study provides ultrastructural and molecular support of Tau role in myelination of PNS. Neuronal axon–Schwann cell interaction(s) are essential for myelination, and myelin maintenance is a dynamic process tightly controlled by axon‐dependent transcription factors such as Krox20 and Oct6 (Murphy *et al*., 1996; Decker *et al*., 2006). In line with previous studies demonstrating an absence of axonal deficits in neurons of *Tau−/−* animals (Yuan *et al*., 2008; Vossel *et al*., 2010), there were no differences in mRNA levels of transcription factors that critically regulate myelination process and/or maintenance, for example, Krox20 and Oct6 (data not shown), suggesting no gross changes in myelin gene regulation of *Tau−/−* Schwann cells. Furthermore, supporting the involvement of Tau and other cytoskeletal elements in myelination process, previous evidence suggests that Tau strongly colocalizes with MBP in distal tips of oligodendrocytes (LoPresti *et al*., 1995; Muller *et al*., 1997), suggesting that transportation and/or local MBP translation may require microtubule cytoskeleton and might be controlled by Tau–Fyn interaction (Klein *et al*., 2002). As recent evidence suggests that Tau is responsible to locate Fyn in spines and Fyn has a well‐described role in myelination of both CNS and PNS (Kramer‐Albers & White, 2011), the above Tau–Fyn–MBP complex could be disrupted in the absence of Tau protein, affecting myelination signaling and process. Indeed, Taiep myelin‐mutant animals exhibit irregular microtubule polarity and display abnormal Tau accumulation and intracellular accumulation of myelin proteins in oligodendrocytes (Song *et al*., 2001), while PNS myelin‐deficient mice also exhibited reduction in Tau and other cytoskeletal protein in their sciatic nerves (Kirkpatrick & Brady, 1994). The above findings suggest that the absence of Tau may impact on Schwann cells’ myelination process/signaling, opening a novel window for further research on role of Tau protein in PNS. Furthermore, in light of the suggested therapeutic potential of Tau reduction against AD, a better understanding of the impact of Tau ablation in both CNS and PNS is of great importance. Our findings highlight the morphological and functional implication of peripheral nerves induced by Tau loss in the precipitation of motor deficits with increasing aging and should be taken into account in the development of future AD therapies.

## Experimental procedures

### Animals

In this study, 4‐ to 6‐ and 17‐ to 22‐month‐old male *Tau+/+* and *Tau−/−* (Dawson *et al*. (2001); C57BL/6 background) were used. Mice were housed 4–5 animals per cage under standard environmental conditions with *ad libitum* access to food and water. All experimental procedures were approved by the local ethical committee and national authority for animal experimentation and were in accordance with the guidelines for the care and handling of laboratory animals, as described in the Directive 2010/63/EU.

### Behavioral tests

Rotarod, wire‐hanging, and open‐field tests were performed. Animals were acclimated to the testing conditions for 60 min before testing. For rotarod test (TSE systems, Bad Homburg, Germany), mice were first trained a constant speed (15 rpm) for 3 days (4 trials per day; 60‐s max trial time; 10‐min resting interval among trials). At fourth day, mice (*N* = 9–11 for each genotype) were tested on an accelerating rod (4–40 rpm, 5 min; four trials); in this case, latency to fall was automatically recorded. For wire‐hanging test, animals were placed on a standard wire cage grid, which was then inverted and at 30 cm height from the table surface. The latency to animal fall was manually recorded (two trials; maximum time of each trial: 120 s). The open field was performed in a square arena (43.2 × 43.2 cm) surrounded by tall perspex walls (Med Associates Inc., St. Albans, VT, USA). Mice were placed in the center of the arena allowed to explore during 10 min. Infrared beams were used to automatically register animals’ movements. We next monitored forelimb strength, hindlimb tonus, hindlimb clasping, negative geotaxis, and gait analysis as previously described (Silva‐Fernandes *et al*., 2014). For all tests, scoring was carried out manually by an experimenter blind to the genotype and age of the animals. Briefly, for the assessment of forelimb (grid) strength, animal was positioned on a metal grid and gently pulled off by its tail (hindlimbs were suspended slightly above the grid), while the animal was gripping a wire using its forelimbs. A scale of four different scores was used by the experimenter evaluating forelimb strength of the animal: 0 (no strength), 1 (light/semi‐effective), 2 (moderate/effective), and 3 (active/effective). Hindlimb tonus was tested by pressing animals’ hindlimb paw (by experimenter index finger) evaluating muscular resistance and classifying into four different categories: 0 (no resistance), 1 (slight resistance), 2 (moderate resistance), and 3 (pronounced resistance). Assessment of hindlimb clasping was performed suspending animals by the tail during 30 s. The hindlimbs were observed, and each mouse was given a score for each trial. Hindlimb clasping was rated based on two classes: 1 (hindlimb splayed outward and away of the abdomen) and 2 (hindlimbs retracted inward, toward the abdomen for at least more than 50% of the testing time). Next, we also performed gait analysis based on footprinting. Briefly, the hind‐ and forepaws of the mice were coated with black and red nontoxic paints, respectively. The animals were allowed to walk along 100‐cm‐long × 4.2‐cm‐width × 10‐cm‐height corridor, with ordinary white paper on the floor of the runway for each run. Each animal was tested to achieve one valid trial. The footprint patterns were analyzed manually for four step parameters (measured in cm): the front‐ and hind‐base width, the footstep uniformity, and the stride length. For the analysis of each step parameter, three values were used based on three consecutive steps.

### Sciatic nerve ultrastructure analysis

Under deep anesthesia [ketamine hydrochloride (75 mg kg^−1^) and medetomidine (1 mg kg^−1^)], sciatic nerves were collected and immediately fixed in 4% glutaraldehyde (in 0.1 m sodium cacodylate buffer, pH 7.4) for 7 days and room temperature and then postfixed in 1% OsO_4_, dehydrated. Finally, the tissue was embedded in epon resin (Electron Microscopy Sciences) and sectioned according to the objective (see below). One‐micrometer transverse sections covering the complete cross‐sectional nerve area were stained with 1% p‐phenylene diamine and mounted on Entellan (Merk). Light microscope (Olympus DP70, Hamburg, Germany) images were then mounted on Photoshop and used for manual calculation of number and density of myelinated fibers per transverse section (4–5 animals per group). For the assessment of degenerating fibers, 16 nonoverlapping TEM photographs (3000×) of counterstained ultrathin sections (60 nm) were used (obtained by JEM‐1400 TEM). The same TEM images were also used for g‐ratio [axon diameter/(axon diameter + myelin thickness)] calculation; more than one hundred fibers were measured per animal. Morphometric analysis was performed by an experimenter blind to the samples provenience.

### *Ex vivo* measurement of compound action potentials

Acutely isolated sciatic nerves from each group (6–8 animals per group) were used for the assessment of A‐ and C‐fibers conduction velocity and compound action potentials as previously described (Pinto *et al*., 2008). Briefly, sciatic nerves were dissected and cleaned from the connective tissue sheath in artificial cerebrospinal fluid. Compound action potentials recordings were made with a Multiclamp 700B amplifier in CC mode and digitized with the Digidata 1440a digitizer using PCLAMP 10 software (Axon Instruments, Sunnyvale, CA, USA). Signals were low‐pass‐filtered at an effective corner frequency of 16 KHz and sampled at 50 KHz. Fibers were stimulated at 60 μs, and conduction velocities were calculated for the first compound action potentials peak; total areas were calculated using CLAMPFIT software (Axon Instruments). Electrophysiological recordings and analyses were performed by an experimenter blind to the provenience of the tissue.

### *In vivo* electrophysiological measurements

The *in vivo* approach allowed the acquisition of electroneuromyographic recordings in a noninvasive manner as described previously (Petit *et al*., 2014). Briefly, 5–6 mice per group were anesthetized with a mixture of ketamine (75 mg kg^−1^; Imalgene^®^, Merial, France) and medetomidine (1 mg kg^−1^; Dormitor^®^, Pfizer, New York, NY, USA) i.p. administered. Temperature was monitored and maintained at 37 °C by a homeothermic blanket (Stoelting, Dublin, Ireland). A concentric bipolar platinum/iridium–stainless steel stimulation electrode (WPI, Worcester, MA, USA) was used to deliver 0.1 ms square pulses to the tibial nerve at the ankle, through a small skin incision. The ground stainless steel electrode was inserted at the base of the tail. Compound muscle action potentials (CMAPs) were recorded from the plantar muscles of both hindpaws individually through stainless steel electrodes (diameter: 0.28 mm; Science Products, Hofheim, Germany) inserted in the plantar muscle and subcutaneously in the third toe. Signals evoked by 1‐mA electrical stimulation at 0.1 Hz were amplified, filtered (3–3000 Hz, LP511 Grass Amplifier, Astro‐Med, Rodgau, Germany), acquired (Micro 1401 mkII, CED, Cambridge, UK), and recorded using SIGNAL Software (CED). This setup allowed the recording of CMAPs containing (1) stimulus artifact; (2) early nerve action potential; (3) direct muscle response (M‐wave); and (4) monosynaptic reflex response (H‐wave; Fig. 3A, upper panel); the latest was specifically abolished by lidocaine (2%; B Brown, Germany) injected in the vicinity of the sciatic nerve in the mouse thigh in a control experiment (Fig. 3A, lower panel). Averages of five consecutive responses were analyzed to measure amplitudes and area under curve of M‐ and H‐waves to the baseline. The values obtained for each paw were averaged per animal.

### Western blot analysis

*Tau−/−* and *Tau+/+* sciatic nerves were homogenized [10 mm HEPES pH 7.9, 150 mm NaCl, 1 mm EGTA, 1 mm EDTA, 10% glycerol, 1% NP‐40, Complete Protease Inhibitor (Roche, Mannheim, Germany) and Phosphatase Inhibitor Cocktails II and III (Sigma, St Louis, MO, USA)]. After sonication and centrifugation (15 000 g; 10 min; 4 °C), protein contents were estimated by Bradford assay and lysates were electrophoresed on 10% acrylamide gels, and transferred onto nitrocellulose membranes (BIORAD Turbo, Munich, Germany). For detecting MBP levels, membranes were blocked in Tris‐buffered saline containing 5% nonfat milk in TBS‐T before incubation with antibodies against MBP (Serotec, Oxford, UK; 1:500), Tau (abcam, Cambridge, UK; 1:1000), and actin (DSHB, University of Iowa, IA, USA; 1:2000). Antigens were revealed by enhanced chemiluminescence (BIORAD) after incubation with appropriate horseradish peroxidase–immunoglobulin G conjugates (BIORAD). Blots were scanned and quantified using tina 3.0 bioimaging software (Raytest, Straubenhardt, Germany). All values were normalized against actin.

### Statistical analysis

Unless otherwise specified, two‐way ANOVA was used having genotype (*Tau+/+* vs. *Tau−/−)* and aging (adult vs. old) as factors and followed by Tukey post hoc analysis (SPSS, Aspire Software, Armonk, NY, USA). Differences were considered to be significant if p < 0.05.

## Authors’ contribution

SL, AL, VP, VMS, SP, JP, JFO, HLA, and IS performed experiments; SL, AL, MG, VMS, JFO, HLA, and IS analyzed data and contributed with reagents; and IS, NS, and HLA designed the overall study and wrote the manuscript.

## Conflict of interests

None of the authors report competing interests.

## Funding

The work was supported by grants ‘PTDC/SAU‐NMC/113934/2009,’ ‘PTDC/SAU‐NSC/118194/2010,’ ‘SFRH/BPD/97281/2013,’ PTDC/SAU‐NSC/118194/2010,’ ‘SFRH/BPD/80118/2011,’ ‘SFRH/BD/89714/2012’ funded by FCT—Portuguese Foundation for Science and Technology and project DoIT—*Desenvolvimento e Operacionalização da Investigação de Translação* (N° do projeto 13853), funded by *Fundo Europeu de Desenvolvimento Regional* (FEDER) throughout the *Programa Operacional Fatores de Competitividade* (POFC). In addition, this work was also co‐financed by European Union FP7 project SwitchBox (NS) and the Portuguese North Regional Operational Program (ON.2 ‐ O Novo Norte) under the National Strategic Reference Framework (QREN), through the European Regional Development Fund (FEDER).
